# Case Report: The effect of intravenous and oral antibiotics on the gut microbiome and breath volatile organic compounds over one year

**DOI:** 10.12688/wellcomeopenres.17450.1

**Published:** 2022-02-11

**Authors:** Farah Shahi, Sarah Forrester, Kelly Redeker, James P.J. Chong, Gavin Barlow

**Affiliations:** 1 Department of Biology, University of York, UK, York, YO10 5DD, UK; 2 Department of Infection, Hull University Teaching Hospitals NHS Trust, Hull, HU3 2JZ, UK; 3 Experimental Medicine and Biomedicine, Hull York Medical School, University of York, York, YO10 5DD, UK

**Keywords:** microbiome, volatile organic compounds, antibiotics, breath metabolites, resistance, antimicrobials

## Abstract

Background: Antimicrobial resistance (AMR) is a global concern and better understanding of the gut microbiome, a known ‘amplifier’ of AMR, may allow future clinicians to tailor therapy to minimise this risk and offer a personalised medicine approach. To examine the gut microbiome, patients are required to provide faecal samples; more convenient and cheaper solutions need to be found.

Methods: As part of a pilot study looking at how routes of administration affect the gut microbiome in NHS patients undergoing routine clinical management for infections, we hypothesised that effects on the gut microbiome varied with the route and metabolism of antibiotic used, and these changes may be reflected in breath metabolites. We present a case report of a patient with an unusual clinical history, alongside breath metabolite and gut microbiome data taken before, during and after antibiotic therapy over a period of one year.

Results: We noted a shift in the dominant *Bacteroides* strain in the patient’s gut microbiome between pre- and post-therapy samples, along with an alteration in the composition of breath metabolites.

Conclusions: This study provides a framework for similar future work and highlights the need for further research on the relationships between changes in microbial gut communities and antimicrobial exposure, patient clinical status, and the metabolites of human breath.

## Introduction

Scientific developments have enabled the possibility of personalised medicine at this time more than any other. In an age where antimicrobial resistance (AMR) is a global concern, insights into the gut microbiome, a known ‘amplifier’ of AMR ^1^, may in the future aid clinicians in tailoring therapy for their patients to reduce this and other health risks. However, for patients, providing faecal samples can be an uncomfortable experience ^2^, and the cost of sequencing individual patients’ gut microbiomes, both in terms of time and resource, still remains prohibitive. Increasingly, scientists and clinicians are searching for more convenient options for patients, including the use of rectal swabs ^3^.

As part of a pilot study looking at how routes of administration affect the gut microbiome in NHS patients undergoing routine clinical management for infections, we hypothesised that: i) the effects on the gut microbiome would vary with the spectrum of antibiotic used, the route of delivery, and the metabolism of the drug and ii) related effects may be identifiable in breath metabolites.

Breath analysis, or breathomics, is an exciting but challenging field of science and medicine. Agreeable to the patient due to its non-invasive nature, it has great potential for individualized point-of-care diagnostics and screening ^4^. Volatilomics, the study of Volatile Organic Compounds present in breath, is a systemic analysis, combining metabolic signals from the entire body through the blood-lung interface as well as the gut microbiota ^5^.

Here we present a case report of a patient with an unusual clinical history, alongside breath metabolite and gut microbiome data taken before, during and after antibiotic therapy over a period of one year. There are few such case reports in the literature to date.

### Case report

Patient A, a 57-year-old Caucasian male, presented to clinicians at Hull University Teaching Hospitals NHS Trust with an 11-year history of biliary system infection. In 2006 he had undergone a laparoscopic cholecystectomy for cholecystitis; this was complicated by a ‘dropped’ gallstone (a stone that remains in the abdominal cavity after surgery), subsequent recurrent hepatic infections and abdominal wall fistulation. Multiple surgical and radiological attempts at stone retrieval and drainage were eventually successful in 2016. However, six months later he continued to suffer recurrent infections and a CT scan in July 2017 revealed a persistent perihepatic abscess and hepatic-cutaneous fistula devoid of visible calculi. Consequently, he was referred to the Outpatient Parenteral Antimicrobial Therapy (OPAT) service for consideration of prolonged antimicrobial therapy.

On examination of the patient, a fistula was evident in the right upper quadrant, which correlated with a sinus tract on his CT. Baseline physiological observations were normal. Blood tests showed a C-reactive protein (CRP) of 17 mg/L (normal range 0 to 8 mg/L) and total white blood cell count of 7.9 × 10 ^9^/L (normal range 4.0 to 11.0 × 10 ^9^/L) but were otherwise unremarkable. Based on the patient’s relevant clinical microbiology results ^6^, applicability to OPAT usage and longer-term tolerability, a decision was made to start intravenous (IV) Ertapenem 1g once daily. This was continued for 38 days, followed by oral Ciprofloxacin 750mg every 12 hours for 23 weeks.

Prior to starting therapy, Patient A was enrolled into the aforementioned pilot study following informed, written consent. Ethical approval for the study was granted by the HRA, Leicester REC 16/EM/0345 and the University of York Biology Ethics Committee. In total, seven faecal and seven breath samples were provided by Patient A between September 2017 and August 2018. The first samples were taken prior to starting antibiotic therapy; further sampling times are listed in Table 1.

**Table 1.** Sampling Schedule for Patient A relative to antibiotic treatments. IV = intravenous, PO = per os (by mouth), OD= omni die (every day - implied to be once per day), BD = bis die (twice a day).

Sample Name	Antibiotic	Timing of Sample
Pre-antibiotics	N/A	Baseline samples
Sample 1	Ertapenem 1g OD IV	8 days into therapy
Sample 2	Ertapenem 1g OD IV	14 days into therapy
Sample 3	Ertapenem 1g OD IV	21 days into therapy
Sample 4	Ciprofloxacin 750mg BD PO	5 days into oral therapy
Sample 5	Ciprofloxacin 750mg BD PO	78 days into oral therapy
Post-antibiotics	N/A	122 days without therapy

## Results

### Sequencing and assembly of faecal specimens

Microbial DNA extracted from stool samples was subjected to long- and short-read DNA sequencing. The final polished assembly comprised of 9,554 contigs, N50 56,726bp and a total length of 159,132,373bp (see 6 for read statistics). 156,292 genes were identified using Prodigal version 2.6.3, within the anvi’o pipeline ^7^. 71.7% of contigs were annotated, with 1,295 unique taxon names. CONCOCT annotation resulted in 81 bins from the data, and the manual tree-based approach resulted in 13 bins ^8^. The manually curated clusters were favoured over those generated by CONCOCT due to 48 of the 81 bins not being resolved into a genus annotation. This is because annotation is based on the most abundant annotation within the bin, and so the most abundant genera may be unknown, and account for less than 30% of the contigs within the bin. CONCOCT bin annotations are provided ^6^. All manually curated bins were annotated, with 8 of 13 being annotated as *Bacteroides*. The annotation and semi-quantitative abundance relative to the stage of treatment are provided ^6^.

**Figure 1.**
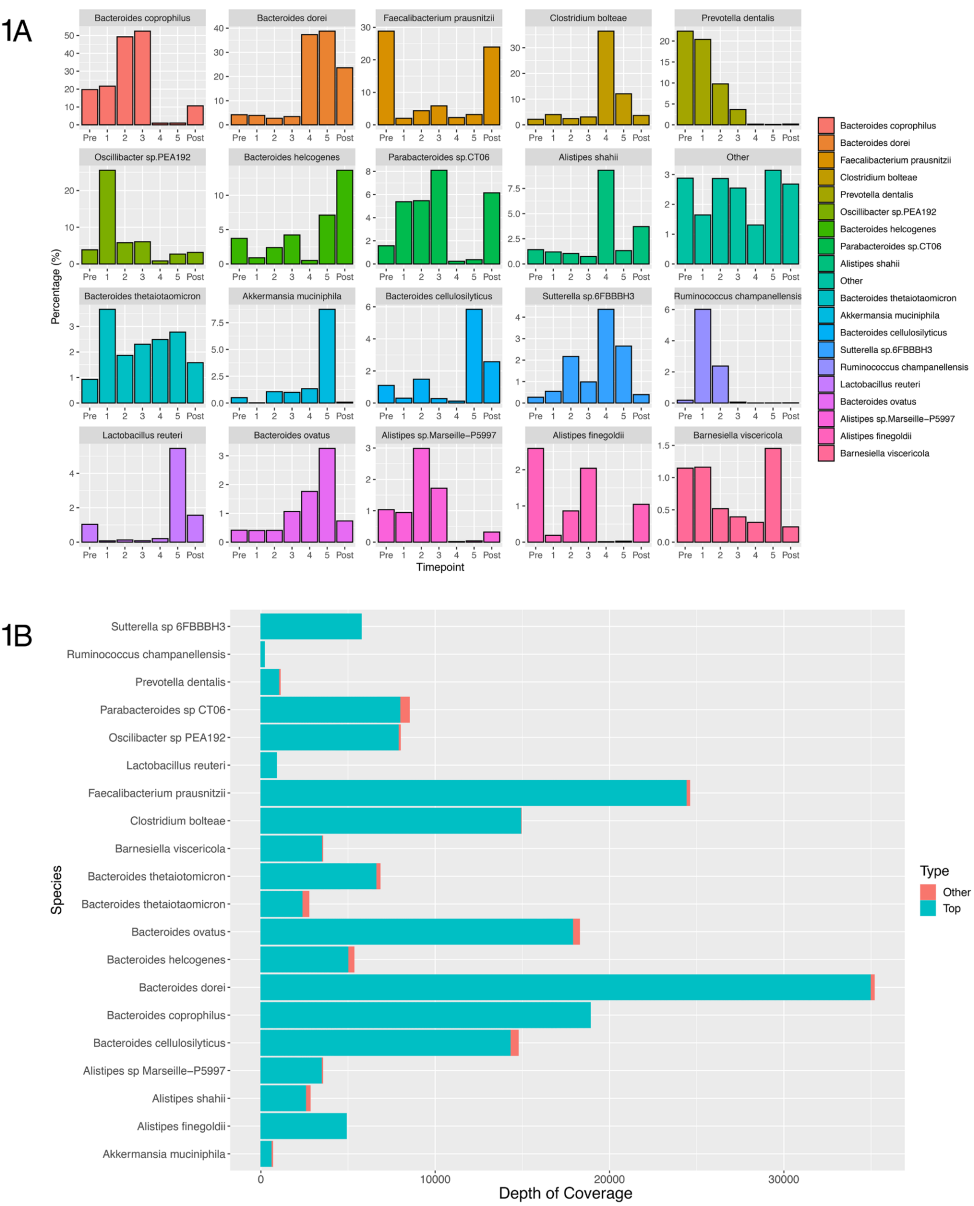
( **A**) Barplots showing the relative abundance of each of the top 19 most abundant MAGs generated by CLUSTARD, with the most abundant species identified in the binned contigs given as the label. All other identified species are contained within the other bin. Abundances are based on coverage per sampling time point. ( **B**) Shows the number of bases per species that are predominant in each cluster, that are not contained within these top 19 MAGs. They show that the number of bases outside of these top 19 bins are minimal.

Neither of the previous strategies were able to resolve the data into single genome bins, and both resulted in bins that were either overfilled or highly contaminated with contigs from other genomes, preventing resolution of the data to species level. A custom analytical pipeline written in-house (CLUSTard, Cansdale *et al.*, in prep) was able to identify 19 metagenome assembled genomes (MAGs) that we assumed represented different species as shown in Figure 1A, which constituted over 97% of the patient’s microbiome. The abundance of the additional annotated species is summarised within the “Other” bin within Figure 1A. These are cumulative abundances taken from all clusters containing the same species annotation, and so some of the species are explained through multiple bins. However, different MAGs with the same species identity have a similar trajectory for the majority of bases within that bin. Contigs that have the same annotation as any of the top 19 MAGs but are not included within Figure 1A are minimal, as shown in Figure 1B. These contigs had both a different trajectory and only represented a small percentage of the total bases of that bin. The full results showing the abundance per bin and per contig for the entire assembly are available as extended data ^6^.

The two most abundant species throughout the time course are *Bacteroides coprophilus* and *Bacteroides dorei* ( Figure 1A). *Bacteroides coprophilus* is high in abundance during pre-treatment and the course of IV Ertapenem, whereas *Bacteroides dorei* is low in abundance during IV Ertapenem, but becomes highly abundant during oral Ciprofloxacin therapy, and following treatment.

Comparison of the MAGs used in the assembly for the species in Figure 1A compared to the National Center for Biotechnology Information (NCBI) reference genomes for the same species suggests that we do not have complete genomes for all of the top 19 most abundant species. The size of the assembled genomes relative to the NCBI reference for *Oscillibacter* (160%), *Bacteroides dorei* (124%), and *Faecalibacterium prausnitzii* (160%) suggest mis-assemblies that make the genomes larger than the expected size. However, the size of the assembled *Bacteroides coprophilus* genome is double that expected, which suggests that we could have two strains of *Bacteroides coprophilus* that have been assembled. There are two high quality MAGs that are singletons (one contig assembled genomes) that are annotated as *Bacteroides coprophilus*, Cluster_singleton2 and Cluster_singleton4, which are genome sized. However, they have the same trajectory suggesting that they respond to antibiotic treatment in the same way.

Three species, *B. helcogenes*, *B. cellulosilyticus* and *Lactobacillus reuteri,* are significantly increased in the post-treatment samples ( Figure 1A). *Faecalibacterium* is strongly suppressed during antibiotic treatment but recovers after treatment stops. Levels of *Prevotella dentalis* were higher prior to treatment with IV Ertapenem, and continued to reduce during oral Ciprofloxacin, remaining at low levels post-treatment. *Parabacteroides* is also more abundant during IV Ertapenem use and post-treatment but is suppressed during oral Ciprofloxacin treatment. There are three *Alistipes* species: two species are suppressed during oral Ciprofloxacin treatment, and displaced by *Alistipes shahii*, which is the most abundant during this phase. Following treatment there is a more equal mix of all three species, with *Alistipes finegoldii* and *Alistipes* sp. Marseille not recovering to pre-treatment levels but *Alistipes shahii* remaining higher ( Figure 1A). *Ruminococcus champanellensis* significantly increased during treatment with IV Ertapenem but reduced during oral Ciprofloxacin therapy, remaining low post-treatment. Finally, three species were noted to become particularly abundant in the final sample taken on antibiotics: *Lactobacillus reuteri*, *Akkermansia muciniphila* and *Bacteroides ovatus*.

Resistance genes were identified in both the *B. coprophilus* and *B. dorei* reference genomes, and the most abundant MAGs in these two species. As suggested by the assembly size being double the size of the NCBI reference genome, the presence of two strains of *B. coprophilus* is also suggested by the identification of different resistance genes present within different *B. coprophilus* MAGs ^6^. 75% of all *B. coprophilus* annotated bases were split between four MAGs, Cluster_contig0015 (15%), Cluster_contig0024 (19%), Cluster_singleton2 (26%) and Cluster_singleton4 (14%). The remaining bases are split across 14 lower abundance and quality clusters. 67% of all *B. dorei* annotated bases are within the MAG Cluster_contig0017. The remaining bases are split across > 20 other lower abundance and quality clusters. Cluster_contig0017 was used as the representative MAG for *B. dorei* for gene resistance annotation and the four aforementioned MAGs were used for *B. coprophilus*.

In *B. coprophilus* (more abundant during pre-treatment and IV Ertapenem) MAGs Cluster_singleton4 and Cluster_contig0015 have lost/have absent all three resistance genes adeF, tetQ and ErmF present in the NCBI reference. However *B. coprophilus* MAGs Cluster_singleton2 and Cluster_contig0024 have lost tetQ and ErmF genes, but have maintained an adeF resistance gene. Reaffirming the suggestion that there are two strains of *B. coprophilus* strains present. In *B. dorei* (more abundant during oral Ciprofloxacin and post-treatment), we observed an Erm 23S ribosomal RNA methyltransferase and the loss of a tetracycline antibiotic resistance gene ^6^.

Three human gut enterotypes have been identified based on the relative abundance of *Bacteroides*, *Prevotella* and *Ruminococcus* in the gut ^9^. This patient identifies as a Type 1 enterotype in both pre and post samples due to the high abundance of *Bacteroides*. *Prevotella* is also identified but in low abundance, and low but higher levels, compared to *Prevotella,* of *Ruminococcus* is also observed. This is shown consistently between different clustering analyses. Despite no change in enterotype, the composition of the pre- and post-treatment microbiota is different. The *Bacteroides* genus remains the most abundant, however *Bacteroides dorei* displaces *Bacteroides coprophilus* and becomes the most abundant species.

### Breath metabolites are atypical and vary in this patient

**Figure 2.**
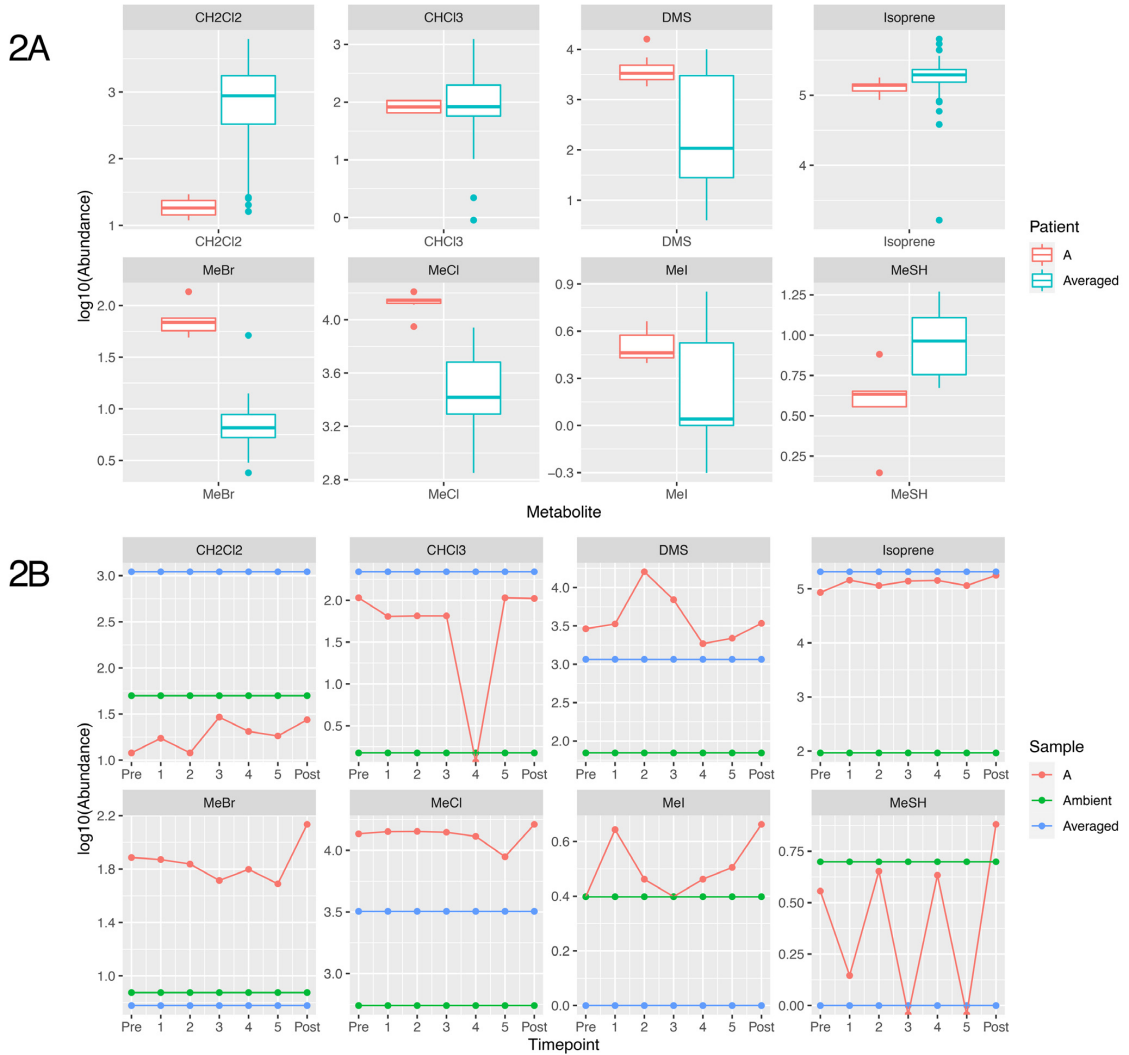
Plot showing the concentration of metabolites identified from breath of patient A. ( **A**) shows a boxplot distribution of metabolite abundance of non-patient samples (n=61 except for MeCl where n=68), compared to the distribution of Patient A’s abundance over the time course. ( **B**) shows the logged 10 abundance of each metabolite compared to the averaged value shown in blue, and the ambient value for the metabolite, shown in green. CH2Cl2 = Dichloromethane, CHCI3 = Chloroform, DMS = Dimethyl sulfide, MeBr = Methyl bromide, MeCl = Methyl Chloride, MeI = methyl iodide, MeSH = Methanoethiol

The breath samples from the patient showed substantial modification from ambient air masses, and for some volatiles the patient exhibited significantly different breath outcomes relative to other sampled and reported individuals. Most prominently different in the patient's breath were the methyl halides and thiol, including methyl chloride, methyl bromide, methyl iodide and methane thiol (CH _3_X, where X = Cl, Br, I or SH and shown as MeCl, MeBr, MeI and MeSH, Figure 2).

In urban air masses methyl halides are found at concentrations of approximately 750, 15 and 5 pptv, respectively ^10^. Humans metabolise these compounds (both in terms of uptake and production) leading to average exhaled breath containing 3500 ± 2400 parts-per-trillion-by-volume (pptv) MeCl, 6.5 ± 11 pptv MeBr and 1.1 ± 1.8 pptv MeI. In contrast, the patient’s average breath profile contained 13,000 ± 2000 pptv MeCl, 73 ± 27 pptv MeBr and 3.3 ± 0.8 pptv MeI. These breath concentrations were significantly different than the average male population for both MeCl and MeBr and consistently more concentrated for all methyl halides. Both methyl chloride and methyl bromide showed consistency from prior to antibiotics and throughout IV Ertapenem therapy (13400 ± 1200 pptv MeCl, 66 ± 9 pptv MeBr) but were significantly reduced during oral Ciprofloxacin (8900 pptv MeCl, 49 pptv MeBr at second sampling, 78 days into oral therapy). Methyl iodide concentrations in the patient’s breath did not notably change over time during antibiotic treatment.

Atmospheric data within urban environments is scarce but our previous breath results suggest that methanethiol (MeSH) is common in human breath at concentrations of 1.7 ± 8.6 pptv. Patient A’s breath was elevated in MeSH relative to healthy human breath, 2.7 ± 2.8 pptv, but not significantly. MeSH results should be viewed with some caution however, due to challenges that derive from storage concerns ^11^. Despite these considerations, MeCl, MeBr and MeSH were all significantly correlated (p < 0.001) with each other (MeCl v.s. MeSH - r ^2^ = 0.49; MeCl v.s. MeBr - r ^2^ = 0.49; MeBr v.s. MeSH - r ^2^ = 0.67). MeI is also significantly correlated (p<0.001) with MeCl, MeBr and MeSH, however correlations are limited (r ^2^ ~ 0.15) between MeI, MeCl and MeSH. Only in the correlation between MeI and MeBr does the correlation coefficient approach 0.5.

Other human-metabolised halogenated compounds showed interesting outcomes in Patient A, including dichloromethane (CH _2_Cl _2_) and chloroform (CHCl _3_). Ambient urban air contains approximately 5600 and 1300 pptv, respectively, while average healthy human breath is generally less concentrated, although highly variable, at 1000 ± 1500 pptv for dichloromethane and 230 ± 660 pptv for chloroform. The patient exhibited uniform, and depleted, concentrations for dichloromethane throughout the study, 20 ± 6 pptv dichloromethane, while chloroform was uniformly low but highly variable, at 95 ± 71 pptv. Concentrations of chloroform showed a strong time, and possibly therefore antibiotic dependency, with starting concentrations near 105 pptv that fell throughout IV Ertapenem to a level below the detection limit (<5 pptv) by the start of oral Ciprofloxacin, returning to approximately the same concentrations as the earliest sample post-treatment (~105 pptv).

The sulfur-containing compound, dimethyl sulfide (DMS) was present in the patient's breath, ranging from 900 to 16000 during the various stages of treatment. While the average breath concentration of DMS (4700 ± 4900 pptv) in patient A was similar to human breath averages (2300 ± 2300 pptv DMS), this was primarily due to the high variability of this data across individuals and populations. DMS concentrations appeared to be treatment dependent, with a substantial increase in DMS during IV Ertapenem therapy (from 2900 to 16000 pptv) before falling during oral Ciprofloxacin to near average healthy human concentrations (~2800 pptv).

Isoprene, the most abundant human metabolite in breath, was similar between the patient (125 ± 23 parts-per-billion-by-volume; ppbv) and the healthy human population (185 ± 205 ppbv). Baseline concentrations of isoprene can vary substantially between individuals (from 80 to 300 ppbv within our healthy controls), but within the established baseline it tends not to vary dramatically, which is what we observed in the patient post-antibiotic therapy when isoprene levels rose to 178ppbv.

### Clinical follow-up

**Figure 3.**
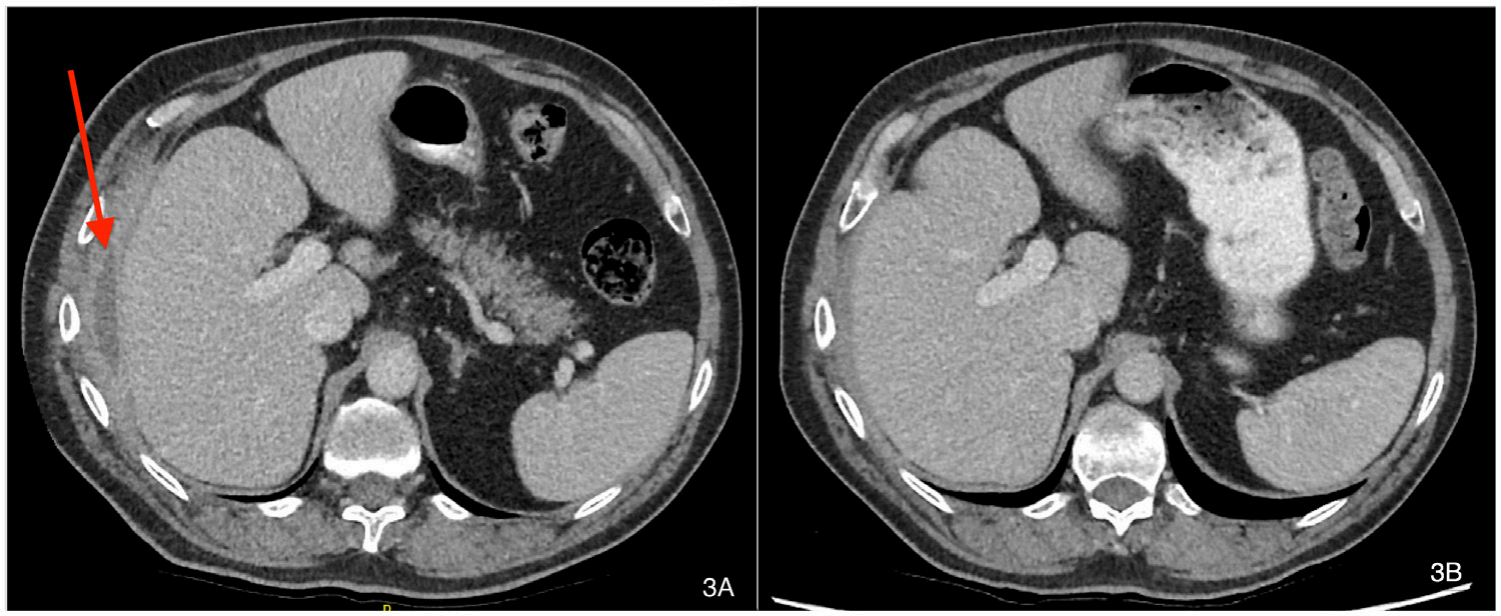
CT imaging before (A) and at 16 weeks (B) of antibiotic therapy (labels added with Mac’s Preview App v11.0).

Patient A symptomatically improved during treatment, which resulted in clinical and radiographical resolution of the hepatic-cutaneous fistula. A repeat CT scan seven weeks into antimicrobial therapy suggested the patient’s infection had not fully resolved, but a further CT scan at 16 weeks of therapy showed no evidence of ongoing infective changes and resolution of the previous peri-hepatic abscess (see Figure 3). He was discharged from clinical follow up in March 2019 with a CRP of 3.1 mg/L and has not suffered recrudescence since.

## Discussion

Understanding and mitigating the consequences of antimicrobial exposure on the human gastrointestinal tract microbiome at an individual level is likely to become increasingly important in the era of personalised medicine, given our increasing understanding of the associations between the nature of an individual’s gastro-intestinal (GIT) microbiome and the risk of a disease state. The GIT microbiome is also recognised as an ‘amplifier’ of antimicrobial resistance and changes in an individual’s microbiome may contribute to this. In this pilot study, we sought to describe the changes occurring in patients’ gut microbiome during IV and oral antibiotic therapy, and assess whether these changes were reflected in breath metabolites.

In Patient A, *B. coprophilus*, a bacterium that has not been associated with human infection, was abundant throughout IV Ertapenem exposure (suggesting the presence of a resistance mechanism), suppressed during oral ciprofloxacin, and abundant again post-therapy. Low GIT exposure is unlikely to be the cause of abundance during Ertapenem therapy as 10% of a 1g dose is excreted in human faeces (Ertapenem 1g Powder for Concentrate for Solution for Infusion, *Electronic Medicines Compendium*, 2020 *).* Meropenem exposure, however, has previously been associated with the emergence of carbapenem resistance in *Bacteroides fragilis*, which is commonly associated with the cfiA metallo-beta-lactamase gene ^12^. Fluoroquinolone susceptibility in *B. fragilis* in the presence of carbapenem resistance has been described in bloodstream infection ^13^, although resistance specifically to Ciprofloxacin in *B. fragilis* strains is generally expected to be high ^14^. However, in Patient A, the abundance of the adeF gene throughout, which has been associated with efflux-mediated resistance to fluoroquinolones and tetracyclines in clinical practice, suggests that this gene was not expressed by *B. coprophilus* during Ciprofloxacin exposure.

In contrast, *B. dorei* was more abundant during oral Ciprofloxacin exposure and post-therapy suggesting susceptibility to Ertapenem and resistance to Ciprofloxacin, in keeping with potential expression of the adeF gene or another mechanism. Furthermore, the loss of ermF and tetQ from each of the most abundant *Bacteroides* spp., along with the subsequent gain of ermG in the prevailing most abundant species, *B. dorei*, highlights the impact of antimicrobial exposure on AMR within the gut microbiome, which may not always be intuitively related to the class of antimicrobial a patient is exposed to ^15, 16^.

*Faecalibacterium prausnitzii* has been proposed as a biomarker of gut health. An important producer of butyrate, studies have demonstrated an association between gut disease and low levels of *Faecalibacterium* in patients with inflammatory bowel disease ^17, 18^. Patient A’s levels of *Faecalibacterium* remained suppressed during therapy, almost completely recovering to pre-therapy levels on discontinuation. A recent study in mice treated with *Faecalibacterium* supplementation and a prebiotic showed clearance of bacterial burden of *Clostridium difficile* and potential reduction in toxin production compared with untreated mice ^19^. Interestingly, Patient A’s levels of *Clostridium* were low before and after antibiotic exposure, but were high at times during exposure to both antibiotics. The patient did not develop diarrhoea while on antibiotic therapy.

*Bacteroides ovatus*, *Lactobacillus reuteri* and *Akkermansia muciniphila* all showed marked increases at time point 5 of antimicrobial therapy (after 78 days of oral Ciprofloxacin). *Akkermansia* has been labelled a “sentinel of gut health” due to its apparent effect of decreasing gut permeability and its low levels in patients with diabetes and obesity ^20– 22^. Similarly, studies of *Lactobacillus reuteri* have proposed possible anti-inflammatory effects ^23, 24^. *Bacteroides ovatus* has been shown to reduce to LPS-mediated inflammation in animal studies and its use as a probiotic continues to be investigated ^25, 26^. Concurrently there were appreciable decreases in the levels of methylated compounds in Patient A’s breath sample. These findings may be associated with the recovery in Patient A’s clinical status. Further research is warranted to investigate whether markers such as these in patients’ breath and/or faecal samples have the potential, in real-time, to indicate whether a review of the need for ongoing antimicrobial therapy is required.

The use of breath samples in diagnosis and management is attractive because it is minimally invasive and acceptable to patients ^27^. Patient A demonstrated distinct differences in his breath samples compared to the average male in previous studies (unpublished; personal communication, Redeker *et al.* 2021). This highlights the individuality of each person’s results ^28^ and the importance of large sample sizes of the population in understanding what may or may not be statistically generalisable in personalised medicine. However, the broad pattern of changes may in fact be more universal, as demonstrated through “electronic noses”: the use of breath metabolites in the diagnosis of human disease to assess patterns of volatile organic compounds (VOCs) associated with specific illnesses ^27, 29^. There is increasing evidence that exhaled breath and the gut microbiome are strongly correlated ^30– 32^. The method of breath analysis we used for Patient A is more amenable to a personalised medicine approach but pattern changes over time may carry more significance than individual VOCs obtained at single timepoints during patient care. The inverse association of chloroform levels and antibiotic therapy may be of interest; if such relationships can be robustly established, it may be possible to monitor aspects of drug therapy at the bedside via the breath, for example, rather than current invasive techniques. 

Most VOCs in Patient A’s breath did not return to starting-point values on completion of therapy, suggesting that microbial/human metabolisms within the patient had been substantially modified during illness recovery and/or antimicrobial exposure. Combined with the shift demonstrated in the dominant *Bacteroides* strain within the bowel, as well as significant differences in the microbial community pre- and post-therapy, this suggests potentially measurable modification of microbial communities in patients receiving prolonged antimicrobial therapy. The relevance of these specific community and metabolism changes to health in the longer-term is yet to be understood, particularly as the shifts may represent a return to a pre- or post-illness healthier state or an adjustment to physiological niches held by certain strains that bear no impact on overall function. After all, Patient A clinically improved and became both subjectively and objectively healthier over time, but it is still possible that persisting changes in Patient A’s microbial community as a result of prolonged antimicrobial exposure may have longer-term health implications. 

The complex interplay between different bowel flora *in vivo* in humans remains an important driver for ongoing research of the gut microbiome. Limitations of this pilot project include the likelihood that breath metabolites may be affected by microbiomes or human metabolisms other than those within the gut; for example, the oral and lung microbial communities. However, this study provides a framework for similar future work and highlights the need for further research into the relationships between changes in microbial gut communities during antimicrobial exposure, AMR colonisation and subsequent infection risk, monitoring of patient clinical status, and the metabolites of human breath.

## Methods

### Sample collection

Faecal samples were aliquoted on the day of collection and stored at -80°C. Breath samples were collected in Tedlar bags, protected from light, and transferred into electropolished, stainless steel canisters (LabCommerce, Torrence, CA) via an Ascarite™ trap on the day of collection ^10^.

### DNA extraction, library preparation and sequencing

Microbial DNA was extracted from each faecal sample within 1–18 days post-collection, using a QIAmp PowerFecal DNA Kit. For quantitation purposes, uniquely barcoded Illumina sequencing libraries were prepared from all samples and timepoints using the NEBNext Ultra II FS DNA library prep kit for Illumina (New England Biolabs) according to manufacturer’s guidelines. For each sample 150 ng starting DNA was subjected to a 13 minute fragmentation time, and 4 cycles of PCR amplification using NEBNext multiplex oligos for Illumina (unique dual index primers; NEB). Sample libraries were pooled at equimolar ratios and subjected to paired end 150 bp sequencing on an Illumina HiSeq 3000 (University of Leeds Next Generation Sequencing Facility). Illumina reads were adapter trimmed with cutadapt ^33^ 1.18 with option -a AGATCGGAAGAG to match the Illumina universal adapter.

Long fragment DNA sequencing was performed using an Oxford Nanopore Technologies’ PromethION as part of a larger experiment where for each patient separately, equal quantities of DNA were pooled from each timepoint, and subjected to bead cleaning using an equal volume of AMPure XP beads (Beckmann Coulter). 1.5 ug total DNA from each patient was used in library preparation using the ONT ligation sequencing kit (SQK-LSK109) with native barcode expansion pack (EXP-NBD103). The DNA was subjected to DNA repair/A-tailing using the NEBNext ^®^ Ultra™ II End Repair/dA-Tailing Module, with additional NEBNext FFPE repair enzyme (NEB), with sequential incubations for 30 minutes at 20 °C and then 65 °C. Following clean up with 0.9 volumes AMPure XP beads, a unique barcode adapter was ligated to the prepared DNA from each patient using NEB Blunt/TA ligase mastermix. An additional AMPure clean-up was performed before pooling approximately equimolar quantities of barcoded sample from each patient. The sequencing adapter from EXP-NBB103 was then ligated to the fragment ends using the NEBNext Quick ligation module. A final AMPure XP bead clean up, including two washes with ONT long fragment wash buffer (LFB) was performed prior to library quantitation using the Qubit fluorimeter (Thermo Fisher). The library was loaded onto a FLO-PRO002 R9.4.1 flow cell, following manufacturer’s guidelines, and sequenced for 56 hours.

### Sequence assembly

Sequencing data was basecalled with Guppy 1.8.3, and demultiplexed with Porechop 0.2.3. Nanopore reads were assembled with canu ^34^ 1.8 using parameters genomeSize=100m, corMinCoverage=0, corOutCoverage=all, corMhapSensitivity=high. The canu assembly was polished with the nanopore reads using medaka 0.7.1 and with the Illumina reads using Pilon ^35^ 1.23, running Pilon three times, aligning reads to the assembly with bwa ^36^ mem 0.7.17 and indexing and sorting read alignments with SAMtools ^37^ 1.9. All tools were run with default options unless otherwise stated.

### Sequence analysis

The assembly was filtered to remove contigs less than 1000bp, (see extended data ^6^ for contig length distribution) before being annotated and clustered using the anvi’o metagenomic workflow using the centrifuge nt database ^7, 38^. Clustering was performed using both an automated method using CONCOCT and manually using hierarchical clustering of contigs ^8^. Taxonomy was generated based on the contigs, and the abundance of these taxon through the coverage of the short reads mapped to the assembly using bwa mem 0.7.17 ^36^.

The metagenomic clustering pipeline CLUSTard (public publication pending) was used to generate clusters of contigs based on mapped short read abundance, using a kraken2 database. Pearson coefficient correlation values were varied to determine percentage similarity in trajectory between contigs within a bin over time. Values between 0.997 and 0.8 were used to assess clustering, with 0.997 used in further analyses.

Contigs from both highly abundant clusters in *Bacteroides coprophilus* (Cluster_singleton2, Cluster_singleton4, Cluster_contig0024 and Cluster_contig0015) and the most abundant cluster in *Bacteroides dorei* (Cluster_contig0017), were annotated using the CARD: RGI database ^39^ to identify antimicrobial resistance genes that could be responsible for their trajectories throughout treatment. The NCBI reference genomes for *Bacteroides salanitronis* (CP002530.1) and *Bacteroides dorei* (CP008741.1) were used to identify expected resistance genes in the assembly and identify subsequent losses or gains in the contigs within these MAGs. *Bacteroides salanitronis* was used as a reference because it is closely related, and *Bacteroides coprophilus* is not present currently in this database. These MAGs were mapped to the NCBI references of both *Bacteroides Coprophilus* and *Bacteroides Salanitronis* using default options in minimap2 ^40^. There was no significant difference in the percentage of sequence mapping to these references, which we were satisfied meant that the genomes were similar enough that mapping to *Bacteroides Salanitronis* using the CARD RGI database would not impact on the resistance genes identified. 

### Breath volatile metabolite analysis

Tedlar bags provide ease of sampling for patients but are unsuitable for long term storage of volatiles. To ensure stable long-term storage we transferred the breath sample from the Tedlar bag to high-quality stainless steel canisters (electropolished prior to single welding) on the same day. Prior to sample transfer from the Tedlar bags the canisters were evacuated (<0.1mbar pressure). To ensure stability and sensitivity of analysis each sample passed through an Ascarite trap, driven by the pressure differential between the Tedlar bag and canister. The volatiles within this study have been shown previously ^41^ to be stable in these canisters for over two weeks.

Canister-bound breath samples were concentrated onto a liquid nitrogen condensation trap through pressure differential, driven by a two-stage rotary pump, then run on a Gas Chromatograph-Mass Spectrometer (GC-MS) consisting of a Restek© PoraBond Q column (30m, 0.32mm ID, 0.5-μm thickness) within a HP 5972 MSD, running in selective ion mode, within six days of the date of collection ^41^. We used CFC-11 as an internal standard since it is ubiquitous and constant in the atmosphere and there are no known metabolisms that produce or consume it within the human body.

Concentration calibrations were performed using purchased, low-concentration (ppbv) standards (BOC Specialty Gases; CFC-11, CFC-12, CFC-113, methyl halides, DMS) directly injected into the condensation system. Within our controlled temperature and volume trap, injection of a limited pressure of high concentration gas (the standards) mimics the same total amount of a greater pressure injection of a low concentration sample (breath samples). Accuracy of these standards for breath sample concentrations were ±10%. Alternatively, serial dilutions of headspace volatiles from neat standards were used (as in isoprene, methane thiol). These standards were less reliable than the prepared standards because of the small scale on which the system is operated as well as differences in reported vapor pressures for each compound. We calculated accuracy for quantification of samples based on these standards to be ±20%.

## Data availability

### Underlying data

Whole metagenome sequencing, using long and short reads for the assembly of a gut microbiome. Accession number PRJEB44880; https://identifiers.org/ena.embl:PRJEB44880

### Extended data

Open Science Framework: The effect of intravenous and oral antibiotics on the gut microbiome and breath volatile organic compounds over one year: a case report. https://doi.org/10.17605/OSF.IO/NQ7ZA ^6^

Data are available under the terms of the Creative Commons Zero "No rights reserved" data waiver (CC0 1.0 Public domain dedication).

## Consent

Written informed consent for publication of their clinical details and images was obtained from the patient.
