# Supplementary material for: Case Report: The effect of intravenous and oral antibiotics on the gut microbiome and breath volatile organic compounds over one year
Source: Wellcome Open Res. 2022 Dec 5;7:50. Originally published 2022 Feb 11. [Version 3] doi: 10.12688/wellcomeopenres.17450.3 (PMC9975432; doi:10.12688/wellcomeopenres.17450.3)
Supplement: Peer review history

# Peer review: Reviewer response for version 3

**DOI:** 10.21956/wellcomeopenres.20688.r53574

**Published:** 2022-12-20

**Authors:** Mark P. Nicol

**Affiliations:** 1 Division of Infection and Immunity, School of Biomedical Sciences, The University of Western Australia, Perth, WA, Australia

Thank you to the authors for your detailed response to my comments. I have no further comments.

Are enough details provided of any physical examination and diagnostic tests, treatment given and outcomes?

Partly

Is the case presented with sufficient detail to be useful for other practitioners?

Partly

Is sufficient discussion included of the importance of the findings and their relevance to future understanding of disease processes, diagnosis or treatment?

No

Is the background of the case’s history and progression described in sufficient detail?

Partly

Reviewer Expertise:

Clinical microbiology, microbiome, tuberculosis

I confirm that I have read this submission and believe that I have an appropriate level of expertise to confirm that it is of an acceptable scientific standard.

# Peer review: Reviewer response for version 2

**DOI:** 10.21956/wellcomeopenres.19790.r50987

**Published:** 2022-07-11

**Authors:** Pisut Pongchaikul

**Affiliations:** 1 Chakri Naruebodindra Medical Institute, Faculty of Medicine Ramathibodi Hospital, Mahidol University, Samut Prakarn, Thailand; 2 Institute of Infection, Veterinary and Ecological Sciences, University of Liverpool, Liverpool, UK

Thank you to the authors for your consideration. The current version has been significantly improved.

Are enough details provided of any physical examination and diagnostic tests, treatment given and outcomes?

Yes

Is the case presented with sufficient detail to be useful for other practitioners?

Yes

Is sufficient discussion included of the importance of the findings and their relevance to future understanding of disease processes, diagnosis or treatment?

Yes

Is the background of the case’s history and progression described in sufficient detail?

Yes

Reviewer Expertise:

Microbiology, Antibiotic resistance

I confirm that I have read this submission and believe that I have an appropriate level of expertise to confirm that it is of an acceptable scientific standard.

# Peer review: Reviewer response for version 2

**DOI:** 10.21956/wellcomeopenres.19790.r50986

**Published:** 2022-06-22

**Authors:** Mark P. Nicol

**Affiliations:** 1 Division of Infection and Immunity, School of Biomedical Sciences, The University of Western Australia, Perth, WA, Australia

Thank you for your clarifications to my specific comments. However, I don't believe that you have responded to my primary query regarding the association between the metabolome and the microbiome. I have copied my original query related to this below:

 The overall rationale for this study was, as I understand, to assess (in a pilot study) whether exhaled breath metabolites could be used as a proxy for gut microbial profiles. However, no analysis explores the association between gut taxa and the measured metabolites. This is surely a missed opportunity, and reduces the impact of this work. 

 Also, some reflection on the metabolic activity of the gut taxa identified, and their role in producing or modifying the metabolites measured would surely be important. Since whole metagenome sequencing was done it should be possible to do functional analysis to interrogate this question.

Are enough details provided of any physical examination and diagnostic tests, treatment given and outcomes?

Partly

Is the case presented with sufficient detail to be useful for other practitioners?

Partly

Is sufficient discussion included of the importance of the findings and their relevance to future understanding of disease processes, diagnosis or treatment?

No

Is the background of the case’s history and progression described in sufficient detail?

Partly

Reviewer Expertise:

Clinical microbiology, microbiome, tuberculosis

I confirm that I have read this submission and believe that I have an appropriate level of expertise to confirm that it is of an acceptable scientific standard, however I have significant reservations, as outlined above.

# Response

**Published:** 2022-11-29

**Authors:** Farah Shahi

**Affiliations:** University of Sheffield, UK

Many thanks for your comments and your time, and apologies for the delay in responding. Primarily this pilot study was to look at how routes of of administration affect the gut microbiome in NHS patients undergoing clinical management for infections, and to assess the feasibility and suitability of such a study in these patients. As part of this work we wanted to explore our hypothesis that related effects may be seen in breath metabolites. Unfortunately, answering this question fully and completing a full functional analysis remains outside the scope of this small project and case study, particularly as this patient’s breath metabolites appear atypical. However, we accept your point that our exploration of these data is not explicitly stated within the analysis/discussion. We have therefore made some changes to the end of the breath results section that further explains some of our inferences in the discussion as well. For your information, we have also included some additional thoughts below: 

 During our analysis, we conducted a PCA (not included for the reasons discussed forthwith) and other than what we see and describe in the text already (that dorei and coprophilus are replacing each other), we were mostly unable to see direct correlations between the metabolites and the metagenome. However, we did see a negative correlation between methyl chloride and a number of bacteria (see updated end of breath results section). 

 There may be several reasons for a relationship between the breath and faecal metabolic data not being more clear. Firstly, the analysis was metagenomic, not transcriptomic, so there may have been dead biomass seen in the analysis that shrouds the actual changes occurring; secondly, faecal samples may not be fully representative of the entire gut microbiome and there may therefore be alterations seen in breath that add to aspects of the overall picture but that do not directly correlate with faecal data; and thirdly, we measured several metabolites but not a whole metabolome and we therefore might not have seen more obvious trajectories in line with the changing of the organisms present that may occur if the whole metabolome was analysed. Within the discussion we have attempted to draw inferences regarding this patient’s breath data and their microbiome composition over the course of therapy, for example regarding Bacteroides (ovatus), but we have now clarified this further in the results section.

# Peer review: Reviewer response for version 1

**DOI:** 10.21956/wellcomeopenres.19296.r48659

**Published:** 2022-02-25

**Authors:** Pisut Pongchaikul

**Affiliations:** 1 Chakri Naruebodindra Medical Institute, Faculty of Medicine Ramathibodi Hospital, Mahidol University, Samut Prakarn, Thailand; 2 Institute of Infection, Veterinary and Ecological Sciences, University of Liverpool, Liverpool, UK

This work investigated both gut microbiome and breath metabolites of the patient who received antibiotic therapy following surgical complication of laparoscopic cholecystectomy. Authors demonstrated the change of gut microbiome together with breath analysis along the treatment. This work emphasized that antibiotic agents caused dysbiosis in the gut which results in metabolic disturbance, represented by different components of the breath.

 Some points are needed to be clarify to improve this piece of work:

- In Figure 1A, are these percentage shown with respect to the total abundance of each species or to the total bacterial species at each time point?
- Stack plot showing the abundance of bacterial species at each time point should be shown to illustrate the dynamic of the species.
- Authors mentioned Ciprofloxacin resistance and investigated genes that are associated with the resistance. However, common fluoroquinolone resistance genes, such as *qnrA*, *gyrA*, *gyrB, *and *parC*, have not been mentioned. Please describe whether these genes were identified in the *B. dorei.*
- Also, gene associated with carbapenem resistance was not mentioned in *B. coprophilus.*

Are enough details provided of any physical examination and diagnostic tests, treatment given and outcomes?

Yes

Is the case presented with sufficient detail to be useful for other practitioners?

Yes

Is sufficient discussion included of the importance of the findings and their relevance to future understanding of disease processes, diagnosis or treatment?

Yes

Is the background of the case’s history and progression described in sufficient detail?

Yes

Reviewer Expertise:

Microbiology, Antibiotic resistance

I confirm that I have read this submission and believe that I have an appropriate level of expertise to confirm that it is of an acceptable scientific standard, however I have significant reservations, as outlined above.

# Response

**Published:** 2022-05-31

**Authors:** Farah Shahi

**Affiliations:** University of Sheffield, UK

**Many thanks for your comments and for your time. Please see our responses below and within the article.**

 Some points are needed to be clarify to improve this piece of work:

- In Figure 1A, are these percentage shown with respect to the total abundance of each species or to the total bacterial species at each time point?

**It is based on the relative abundance per timepoint, so *Bacteroides coprophilus* at timepoint 3 is 50% of all of the bacteria sampled at time point 3.**

- Stack plot showing the abundance of bacterial species at each time point should be shown to illustrate the dynamic of the species.

**We have added a new Fig 1B to display this, thanks.**

- Authors mentioned Ciprofloxacin resistance and investigated genes that are associated with the resistance. However, common fluoroquinolone resistance genes, such as *qnrA*, *gyrA*, *gyrB, *and *parC*, have not been mentioned. Please describe whether these genes were identified in the *B. dorei.*
- Also, gene associated with carbapenem resistance was not mentioned in *B. coprophilus.*

**Thank you. We have added to the article based on your comments.**

# Peer review: Reviewer response for version 1

**DOI:** 10.21956/wellcomeopenres.19296.r48660

**Published:** 2022-02-21

**Authors:** Mark P. Nicol

**Affiliations:** 1 Division of Infection and Immunity, School of Biomedical Sciences, The University of Western Australia, Perth, WA, Australia

This is an interesting report – more longitudinal analyses such as this are needed. The overall rationale for this study was, as I understand, to assess (in a pilot study) whether exhaled breath metabolites could be used as a proxy for gut microbial profiles. However, no analysis explores the association between gut taxa and the measured metabolites. This is surely a missed opportunity, and reduces the impact of this work. 

 Also, some reflection on the metabolic activity of the gut taxa identified, and their role in producing or modifying the metabolites measured would surely be important. Since whole metagenome sequencing was done it should be possible to do functional analysis to interrogate this question.

 My second major concern relates to conclusions about absolute abundance made on the basis of relative abundance measures, see below.

 Other comments:

 INTRODUCTION

- It probably not possible to test the proposed hypotheses, viz. *“i) the effects on the gut microbiome would vary with the spectrum of antibiotic used, the route of delivery, and the metabolism of the drug, ii) related effects may be identifiable in breath metabolites”* with a single case report. It may be better to reframe the rationale for this study, which was presumably, as a pilot study?
- It is stated that *“Based on the patient’s relevant clinical microbiology results, applicability to OPAT usage and longer-term tolerability, a decision was made to start intravenous (IV) Ertapenem 1g once daily.”* It would be useful to include these results in the text.
- Had no antibiotics been given in the period leading up to the baseline sample collection? This would seem unlikely, given the lengthy delay involved.

METHODS

- For the nanopore sequencing, it is not clear what is referred to here *“as part of a larger experiment where for each patient separately, equal quantities of DNA were pooled from each timepoint”*. There is only one patient. Were all samples from this patient sequenced on the same flow cell?
- Were sequences from all samples pooled for assembly, clustering and taxonomic assignment, or was this done separately for each sample?
- Were ambient air samples collected as controls at the same time of breath collection? This would seem to be key. In figure 2A, what samples are used for the ‘averaged’ values?
- Were breath samples collected in a hospital environment? Are any of the metabolites with high concentrations in this study likely to be found at higher concentration in hospital air vs ambient urban air (which is the comparator used)? 
- Were breath samples collected in the same environment throughout the study? Could this account for some of the differences over time?

RESULTS

- Did the patient take probiotic therapy, which may account for the increase in *Lactobacillus reuteri* following treatment?
- Given the likely mis-assemblies for several key taxa, do the authors think that a single sample-level assembly might assist in teasing this out? 
- Do the different MAGs for *Bacteroides coprophilus* differ in relative abundance by timepoint? If so, how does this relate to the prevalence of the different resistance genes over time? What about for *Bacteroides dorei*?
- I found this sentence very confusing: *“Prevotella is also identified but in low abundance, and low but higher levels, compared to Prevotella, of Ruminococcus is also observed.”*

DISCUSSION

- The increase in *B. coprophilus* relative abundance during ertapenem therapy is interesting. Of course, this is relative, not absolute abundance, which is an important difference. It would be very useful to have some measure of total bacterial load over the course of therapy. Would 16S qPCR help here (even if an imperfect measure)? 
- Given the point above, it is probably not correct to make statements such as *“B. coprophilus, a bacterium that has not been associated with human infection, was abundant throughout IV Ertapenem exposure”* – the persistence (or increase) was in relative abundance and may in fact reflect a decline in absolute abundance if all taxa decrease in abundance, as one might expect during broad spectrum antibiotic therapy.

Are enough details provided of any physical examination and diagnostic tests, treatment given and outcomes?

Partly

Is the case presented with sufficient detail to be useful for other practitioners?

Partly

Is sufficient discussion included of the importance of the findings and their relevance to future understanding of disease processes, diagnosis or treatment?

No

Is the background of the case’s history and progression described in sufficient detail?

Partly

Reviewer Expertise:

Clinical microbiology, microbiome, tuberculosis

I confirm that I have read this submission and believe that I have an appropriate level of expertise to confirm that it is of an acceptable scientific standard, however I have significant reservations, as outlined above.

# Response

**Published:** 2022-05-31

**Authors:** Farah Shahi

**Affiliations:** University of Sheffield, UK

**Thank you for your time and comments. Please see our responses below.**

 INTRODUCTION

- It probably not possible to test the proposed hypotheses, viz. *“i) the effects on the gut microbiome would vary with the spectrum of antibiotic used, the route of delivery, and the metabolism of the drug, ii) related effects may be identifiable in breath metabolites”* with a single case report. It may be better to reframe the rationale for this study, which was presumably, as a pilot study?

**The article text has been updated to clarify this.**

- It is stated that *“Based on the patient’s relevant clinical microbiology results, applicability to OPAT usage and longer-term tolerability, a decision was made to start intravenous (IV) Ertapenem 1g once daily.”* It would be useful to include these results in the text.

**The table from the extended dataset has been moved into the main text as per your suggestion, thank you. **

- Had no antibiotics been given in the period leading up to the baseline sample collection? This would seem unlikely, given the lengthy delay involved.

**Not within 12 months of starting the study. The article text has been updated to clarify this, thanks.**

 METHODS

- For the nanopore sequencing, it is not clear what is referred to here *“as part of a larger experiment where for each patient separately, equal quantities of DNA were pooled from each timepoint”*. There is only one patient. Were all samples from this patient sequenced on the same flow cell?

**Thanks - the article text has been updated to clarify this in the DNA extraction section.** 

- Were sequences from all samples pooled for assembly, clustering and taxonomic assignment, or was this done separately for each sample?

**Thanks - the article text has been updated to clarify this in the Sequence Assembly and Analysis sections.**

- Were ambient air samples collected as controls at the same time of breath collection? This would seem to be key. In figure 2A, what samples are used for the ‘averaged’ values?
- Were breath samples collected in a hospital environment? Are any of the metabolites with high concentrations in this study likely to be found at higher concentration in hospital air vs ambient urban air (which is the comparator used)? 

**Thanks - the legend for Figure 2A has been updated with a reference. For your information though,** **we did not sample for ambient air at the time of the patient sampling, as sampling for all participants was done at different locations, not always in a hospital environment and often in different parts of the hospital. Also, these compounds change in air rapidly, so we can expect a 30% variability from one moment to the next anyway (Low et al., 2003). We, therefore, have a moving background, even if there had been an ambient air sample taken at the same place each time.**

**Variability within the local ambient environment, combined with the fact that the individual’s blood and body were equilibrating with lung volatiles for ~ the previous 15 minutes or so means that an ambient air sample is not particularly informative in this case, and the sampling strategy was constrained by care.**

**However, it is worth noting that methyl chloride, methyl bromide, methyl iodide, and dimethyl sulfide are EXTREMELY high (often over an order of magnitude more than average male concentrations), and not in a way that is matched through any ambient background, internal or external that have been reported. Where the patient is low (isoprene), this is known to be human metabolism (~1,000x ambient background anywhere), so this decrease is notable.**

**Specifically, there are no published records of ambient concentrations of methyl chloride in the parts per billion range. Likewise, dimethyl sulfide concentrations are not seen in the parts per billion range in the ambient atmosphere anywhere. Methyl bromide concentrations do not ever hit hundreds of parts per trillion either, and the key for all of this is that there are no known mechanisms by which they would be released or generated in a hospital or home environment.  Normally, methyl iodide appears to be essentially completely utilized within the human body (as a strong methylating agent it reacts away while in transit), so the fact that it is observed here is indicative of VERY different processes in this individual’s metabolism and something similar can be said for methane thiol.**

 RESULTS

- Did the patient take probiotic therapy, which may account for the increase in *Lactobacillus reuteri* following treatment?

**Thanks - we have addressed this in the introduction now. For your information, participants were asked at baseline regarding their supplement consumption. This participant responded: Cod liver oil capsule every day, orange juice every morning, and regular yoghurts. He did not take probiotics before, or for the duration of, this study.**

- Given the likely mis-assemblies for several key taxa, do the authors think that a single sample-level assembly might assist in teasing this out? 

**Thank you. We are not convinced this would necessarily help. Metagenomes often contain quite a few misassemblies within the contigs, but given that we are looking at the taxonomy based on contig annotations, small errors such as SNPs caused by errors in the long reads, or chimeric reads, should not contribute to bad annotations because they will still match to the nearest taxonomy. Using all of the timepoints together in the assembly means that low abundance taxa are more likely to be represented**

- Do the different MAGs for *Bacteroides coprophilus* differ in relative abundance by timepoint? If so, how does this relate to the prevalence of the different resistance genes over time? What about for *Bacteroides dorei*?

**Cluster_contig0024 and Cluster_singleton2 which have lost and maintained the same genes have a similar trajectory, with a low abundance pre-treatment, peak abundance between timepoints 2 and 3, and then the low abundance following the beginning of the second course of treatment (timepoint 4). This low abundance is maintained even post-treatment. Cluster singleton4 and Cluster_contig0951 both lost all resistance genes. They do not have the same trajectory in relative abundance between timepoints pre- and up to timepoint 3. However, after timepoint 3, both MAGs become very low abundance and do which is maintained even in the post timepoint. Given that the transition between timepoint 3 and 4 represents the transition between different administration routes, it seems like the absence of these resistance genes is contributing to them becoming low abundance following the second treatment course.**

- I found this sentence very confusing: *“Prevotella is also identified but in low abundance, and low but higher levels, compared to Prevotella, of Ruminococcus is also observed.”*

**Thanks, this has been removed as it didn’t add to the point we were making overall regarding the patient’s enterotype.**

 DISCUSSION

- The increase in *B. coprophilus* relative abundance during ertapenem therapy is interesting. Of course, this is relative, not absolute abundance, which is an important difference. It would be very useful to have some measure of total bacterial load over the course of therapy. Would 16S qPCR help here (even if an imperfect measure)? 

**It would, however, 16S abundance still isn’t perfect, and we know that 16S sequencing often results in more skews. This would be less with qPCR versus sequencing, but we don’t think it would add much more to the overall picture.**

- Given the point above, it is probably not correct to make statements such as *“B. coprophilus, a bacterium that has not been associated with human infection, was abundant throughout IV Ertapenem exposure”* – the persistence (or increase) was in relative abundance and may in fact reflect a decline in absolute abundance if all taxa decrease in abundance, as one might expect during broad spectrum antibiotic therapy.

**Thanks - we’ve edited this sentence to clarify it is relative abundance.**
